# Prioritisation of Surgery in the National Health Strategic Plans of Africa: A Systematic Review

**DOI:** 10.1007/s00268-015-3333-9

**Published:** 2015-12-28

**Authors:** Isabelle Citron, Linda Chokotho, Chris Lavy

**Affiliations:** Nuffield Department of Orthopaedics, Rheumatology and Musculoskeletal Sciences, University of Oxford, Oxford, UK; Beit Cure International Hospital, Blantyre, Malawi

## Abstract

**Introduction:**

Disease amenable to surgical intervention accounts for 11–15 % of world disability and there is increasing interest in surgery as a global public health issue. National Health Strategic Plans (NHSPs) reflect countries’ long-term health priorities, plans and targets. These plans were analysed to assess the prioritisation of surgery as a public health issue in Africa.

**Methods:**

NHSPs of 43 independent Sub-Saharan African countries available in the public domain in March 2014 in French or English were searched electronically for key terms: surg*, ortho*, trauma, cancer, appendic*, laparotomy, HIV, tuberculosis, malaria. They were then searched manually for disease prevalence, targets, and human resources.

**Results:**

19 % of NHSPs had no mention of surgery or surgical conditions. 63 % had five or less mentions of surgery. HIV and malaria had 3772 mentions across all the policies, compared to surgery with only 376 mentions. Trauma had 239 mentions, while the common surgical conditions of appendicitis, laparotomy and hernia had no mentions at all. Over 95 % of NHSPs specifically mentioned the prevalence of HIV, tuberculosis, malaria, infant mortality and maternal mortality. Whereas, the most commonly mentioned surgical condition for which a prevalence was given was trauma, in only 47 % of policies. All NHSPs had plans and measurable targets for the reduction of HIV and tuberculosis. Of the total 4064 health targets, only 2 % were related to surgical conditions or surgical care. 33 % of policies had no surgical targets.

**Discussion:**

NHSPs are the best available measure of health service and planning priorities. It is clear from our findings that surgery is poorly represented and that surgical conditions and surgical treatment are not widely recognised as a public health priority. Greater prioritisation of surgery in national health strategic policies is required to build resilient surgical systems.

## Introduction

Diseases amenable to surgical intervention account for 11–15 % of world disability and 5 billion people worldwide lack access to safe affordable surgical and anaesthesia when needed [1–3]. The majority of the surgical disease burden lies in low and middle income countries but only 3 × 4 % of surgery occurs in the poorest third of countries [4]. The recognition of surgery an integral part of a strong health system is gaining traction at a global level with the recent publication of the Lancet Commission for Global Surgery and the inclusion of surgery in the disease control priorities 3 [5, 6].

Most countries have established National Health Strategic Plans (NHSPs) which set out the country’s vision and strategy for development of their health sector to maintain and improve the health of its people [7]. The NHSP defines the nation’s current health situation, lays out a course of action for the achievement of priority targets and defines performance indicators for evaluation of progress. NHSPs are the best available measure of health service planning priorities and therefore can be used to assess the level of priority given to a specific component of health. Strong national surgical plans will be essential in the delivery of resilient surgical systems and universal health coverage, a key post-2015 policy goal [8, 9].

A baseline as to the level of priority given to surgical care planning and provision in Sub-Saharan Africa is yet to be established. This study assesses existing NHSPs for areas relating to the advancement of surgery in African countries. It aims to establish the level of priority given to surgical conditions compared with other health conditions and to identify areas where greater planning is needed if future global surgery targets are to be met.

## Methods

The health plans of the 47 independent Sub-Saharan African countries were searched online in March 2014. Of the 47 countries, 46 had NHSPs available on the WHO Planning Cycle website [10]. Of these, 43 were available in French and English and were included in the study. For those not available on the WHO Planning Cycle website, a separate search was done of the country’s Ministry of Health website and using the online search engine “Google”. This did not return any further results.

To gain an overall sense for the prioritisation of surgery the health plans were electronically searched for mentions of keys terms as seen in Table One. Terms in the glossary were excluded.

Four of the five major domains relating to surgical systems development were assessed [5]. The NHSPS were manually searched for assessments of surgical infrastructure and surgical workforce planning. Service delivery planning was assessed by collating targets and performance indicators for surgical versus non-surgical diseases. Mentions of surgical disease prevalence were used as a measure of information management.

Prioritisation given to surgical financing was not assessed as so few of the national strategic plans contained a budget. Amongst those with a budget, the level of detail was such that disease-specific expenditure could not be meaningfully assessed.

## Results

The 43 policies spanned the years 2002–2030 and had a median of 94 pages (IQR 58-123).

### Mentions

Throughout the 4137 pages analysed, there were a total of 376 mentions of the word root surg* which includes surgery, surgeons, surgical. Of the policies analysed, 19 % (8/43) had no mention of any of the surg* terms. This compared with 3772 mentions of the word HIV or malaria (not including related terms such as antiretroviral drugs or anti-malarials) making a ratio of 10:1 for HIV:surg *.

The assumption was made that more than five mentions of the term surg* was needed to generate a single meaningful surgical policy. 63 % (27/43) of NHSPs contained five or less mentions of any of the surg* terms. This figure of 63 % is more reflective of the overall NHSPs which had no surgical policies then the 19 % which had no mention of the term.

Not one policy contained a single mention of the common general surgical terms hernia, appendicitis/appendicectomy, laparotomy.

Cancer had a higher number of mentions than “surg*” terms at 396 across all the documents. Only 16 % (7/43) of NHSPs had no mention of the term. However, treatment policies related to cancer were mainly non-surgical and were grouped with other non-communicable diseases such as hypertension and diabetes.

Trauma and its related terms were mentioned 239 times across all the documents and at least once in 74 % (32/43) of documents. Of the policies which did mention trauma, only 44 % (14/32) had more than five mentions of the terms which equated to 33 % (14/43) of the total policies. The majority of mentions were in the context of increasing public of awareness of safe driving rather than establishment of trauma registries or provision of emergency services to victims of road accidents or other trauma.

Cesarian and related emergency obstetric terms had 130 mentions in total (see Table 1). 37 % of policies (16/43) had no mention of cesarian section whilst 82 % (35/43) had five or less mentions of the terms.

**Table 1:** Search terms in English and French with total number and frequency of citations

Search terms (English)	Search terms (French)	Total mentions	Policies with no mentions of term (%)	Number of policies with 5 or less mentions (%)
HIV	HIV, VIH	2255	0	2
Malaria	Palu*	1517	2	5
Cancer, malignancy, neoplasm, chemo*	Cancer, néoplasme, chimio*	396	16	56
Surg*	Chir*	376	19	63
Transfusion, blood product	Transfusion, sang*	218	12	58
Trauma, injury, accident, RTA	Trauma*, blessure, accident, AVP	239	23	65
Cesarian, cesarian, C-section, operative deliveries, EMOC, EMONC	Caesa*, Cesa*, SONUC	130	37	81
Cataract, blindness	Cataracte, cécité	61	51	91
Orth*	Orth*	45	70	95
Operating theatre, room, bloc	Bloc opératoire, salle d’opération, opé*	27	37	51
Append*,hernia, laparotomy	Appen*,hernie, laparotomie	0	100	100
Club foot	Pied bot	0	100	100

For simplicity in the remaining text of this paper only the English term will be used to describe the statistics revealed by both English and French search terms

### Infrastructure

72 % (31/43) of policies mapped current health institutions and medical services provided within the country and of these, 61 % (19/31) detailed facilities with existing surgical infrastructure. However, many NHSPs stated that they had insufficient resources to investigate whether these surgical facilities were actually functioning. Surgical services expected at each referral level from rural health clinic to national hospital were laid out in 53 % (23/43) of policies.

### Human resources

All policies included a plan for human resource management. A third of policies (14/43) mentioned plans to increase the number of personnel able to perform surgery. Of these, 57 % (8/14) of the policies planned to increase the surgical skill of general practitioners, 42 % (6/14) mention increasing the recruitment of specialist surgeons and two specifically mentioning recruiting ophthalmic surgeons. The training of other members of the surgical multidisciplinary team such as nurse anaesthetists and theatre technicians was also mentioned in 4 of the 14 policies.

### Service delivery targets

Of the total number of service provision or treatment targets laid out across all the policies, 2 × 3 % (92/4064) related to surgical diseases or improvement of surgical care. A third (14/43) of all health policies analysed did not have a single target which related to the improvement of surgical disease, morbidity or surgical care. With the exception of one policy, there were no targets relating to volumes or morbidity in general surgery. The majority of surgical targets did not have an associated performance indicator to allow evaluation of attainment of the target. This compares with 100 % of policies having both targets and associated performance indicators for reduction in the incidence of HIV and tuberculosis and 98 % of policies having targets for the reduction of the incidence of malaria, maternal mortality and under five mortality.

There were targets to reduce the incidence or mortality from trauma in 53 % (23/43) of the countries. However, there were only four performance indicators allowing evaluation of progress and success in the target. Only 19 % (8/43) of the plans address improving the access to and quality of trauma care, whilst the remainder relate to prevention measures for road traffic and occupational accidents.

There is a target in 26 % (11/43) of countries to start cancer registers, the information from which may help build cancer treatment strategies in the future.

Cervical cancer targets are addressed in 42 % (18/43) of NHSPs and all (18/18) of these policies advocated an increase in screening and national awareness. However, only 22 % (4/18) also mentioned a plan for improving surgical care once cases were detected. A focus towards awareness rather than intervention was apparent across all the cancer targets.

Ophthalmic surgery featured more commonly than other branches of surgery with 30 % (13/43) of policies having a plan and associated performance indicator for either an increase in the number of ophthalmic procedures performed or a reduction in the incidence of trachoma and cataract blindness.

### Information management: disease incidence and prevalence

The incidence of some aspect of cancer was published in 31 % (13/43) of policies with 21 % (9/43) publishing the rate of cervical cancer. The incidence of trauma and its contribution to the burden of mortality and morbidity was published in 47 % (20/43) of the policies. The incidence of cesarian section was reported in 26 % (11/43) of policies. Other surgical diseases where incidence was mentioned included breast cancer (4/43), obstetric fistula (2/43), prostate cancer (2/43) and cardiac surgery (1/43). The incidences of common general surgical disease such as appendicitis, bowel obstruction and hernia were not discussed in any of the policies.

These rates compare with 100 % of policies publishing disease prevalence for HIV and over 95 % publishing prevalence of Tuberculosis, Malaria, infant mortality and maternal mortality. “Neglected Tropical Disease” prevalence statistics were published in 70 % (30/43) of plans, more commonly than general surgery and trauma combined.

## Discussion

Public health policy change is pivotal to public health improvement. The worldwide setting of the millennium development goals (MDG) has had a clear effect on influencing global public health policy with plans to achieve each of the targets featuring heavily on virtually every international NHSP. In the areas of MDG priority, there has been a 32 % reduction in disability adjusted life years (DALYs) between 1990 and 2010 which suggests that public health policy prioritisation is effective in generating public health improvement [11, 12]. As highlighted by this study, health policy for the promotion of surgery in Sub-Saharan Africa remains basic with not a single policy bearing a mention of general surgical disease, 19 % have no mention of surgery and 33 % of policies having no targets relating to surgical care. In the areas of surgery which cross over with the Millennium Development Goals for maternal mortality and HIV, such as cervical cancer and circumcision, there is a mature comprehensive policy which is achieving tangible care improvements [12]. This suggests that given the right stage, surgical policy change can be both attainable and effective.

The methods used in this paper are simple. The number of citations or targets for a particular public health issue in an individual NHSP may not directly correlate with the reality of infrastructure on the ground. However, cumulative analysis of NHSPs gives an overall picture of the current trends in health prioritisation. This is supported by the fact that public health priorities, such as HIV, an area receiving substantial planning, investment and progress, did carry a very high number of mentions and targets. This was in stark contrast to the low mentions of surgical terms.

Dare et al. have explored the reasons why surgery is not currently prioritized on NHSPs [13]. They conclude that competing health priorities, in particular infectious disease, as well as resource constraint and poor framing of the importance of surgery all contribute significantly to the poor current status of surgery.

However, this is a time of opportunity for surgery as a global health issue. The recent inclusion of surgery as part of the WHO disease control priorities 3 and the passage of the World Health Assembly Resolution 68.15 which calls essential and emergency surgical care to be included as an integral part of the Universal Health Coverage means that the notion of surgery as a public health issue is gaining traction. For these opportunities to be realised, clear national health strategic plans must be developed as a road map towards to success.

The Lancet commission on global surgery has a framework of five areas which should be included when building a robust national health surgical strategic plan [5]. These are planning for infrastructure, workforce, service delivery, financing and information management. Policies within each of the five domains of the NHSP must be included, appropriately adapted and developed from the poor baseline determined in this paper. Six key performance indicators to measure the success of surgical policy change have also been promoted in The Lancet Commission and their inclusion in national strategic plans will be paramount [5]. These will allow accountability for current surgical services and the assessment of successes and failures generated by policy change. The National health strategic surgical plan must be written with these targets in mind to ensure they can be delivered to meet these explicit and measurable targets.

## Conclusion

Policy and planning is a cornerstone of public health improvement. Surgery is poorly represented amongst the existing national health plans of Africa. This reflects a low priority status for the development of surgery in the African continent at present. A paradigm shift is needed for surgery to be recognised as a public health issue in Africa.
